# Supplementary material for: Volumetric imaging of fast cellular dynamics with deep learning enhanced bioluminescence microscopy
Source: Commun Biol. 2022 Dec 3;5:1330. doi: 10.1038/s42003-022-04292-x (PMC9719505; doi:10.1038/s42003-022-04292-x)
Supplement: Supplementary file 2 — Supplementary Information [file 42003_2022_4292_MOESM2_ESM.pdf]

Supplementary Material: Volumetric imaging of fast cellular dynamics with deep learning enhanced bioluminescence microscopy

Luis Felipe Morales-Curiel[1], Adriana Gonzalez[1,#], Gustavo Castro-Olvera[1,#], Li-Chun (Lynn) Lin[1], Malak El-Quessny[1], Montserrat Porta-de-la-Riva[1], Jacqueline Severino[2], Laura Battle[2], Valeria Venturini[2,3], Verena Ruprecht[2,3], Diego Ramallo[1], Pablo Loza-Alvarez[1], and Michael Krieg[1,*]

[1] ICFO, Institut de Ciencies Fotòniques, Castelldefels, Spain

[2] Center for Genomic Regulation (CRG), The Barcelona Institute of Science and Technology, Barcelona, Spain

[3] Universitat Pompeu Fabra (UPF), Barcelona, Spain

[*] Correspondence to michael.krieg@icfo.eu

Supplementary Figures

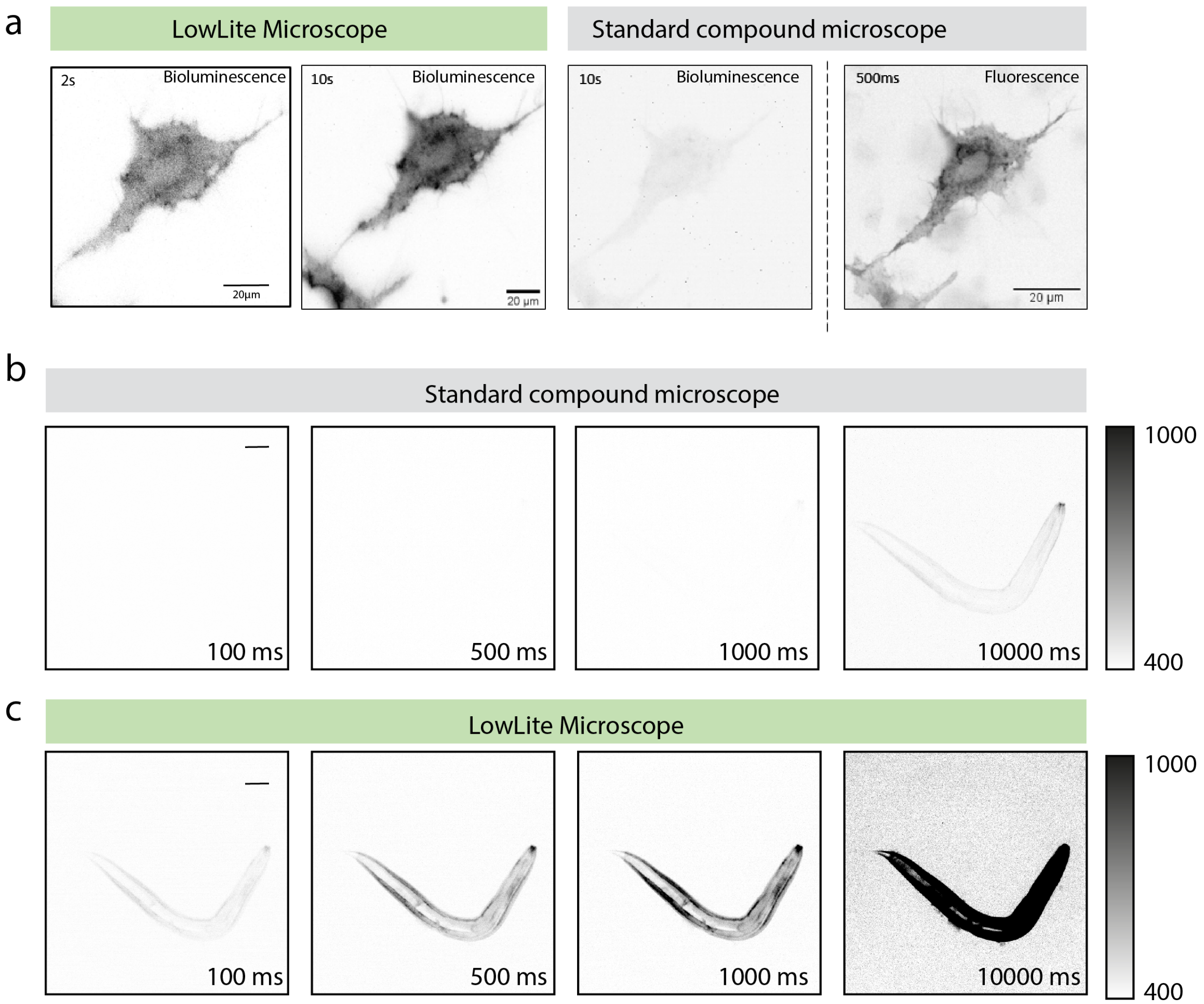

Supplementary Figure 1: Signal of bioluminescent images acquired on commercial compound microscope vs LowLiteScope.
a, Bioluminescent images acquired on Lowlite microscope (22.5x/1.25 SiL, Olympus) compared to fluorescent images taken on a standard epiflourescence microscope (Leica DMi8, 25x/0.95 WI, Leica). Whereas the bioluminescence is barely visible on the standard microscope at 10s exposure times, even though fluorescence is visible, bioluminescence is clearly defined. Scale bar for all images = 50 μm. **b,** Bioluminescent images of transgenic *C. elegans* collected on a Leica DMi8 equipped with a Hamamatsu Orca Flash V3 through a 10x NA 0.31 objective lens with binning 2. All images are scaled between 400-1000 counts. Scale bar for all images = 50 μm. **c,** The same sample was imaged on the LowLiteScope equipped with a Hamamatsu Orca Fusion with a nominal magnification of 15x NA 1.25 objective lens with binning 2. All images are scaled between 400-1000 counts.

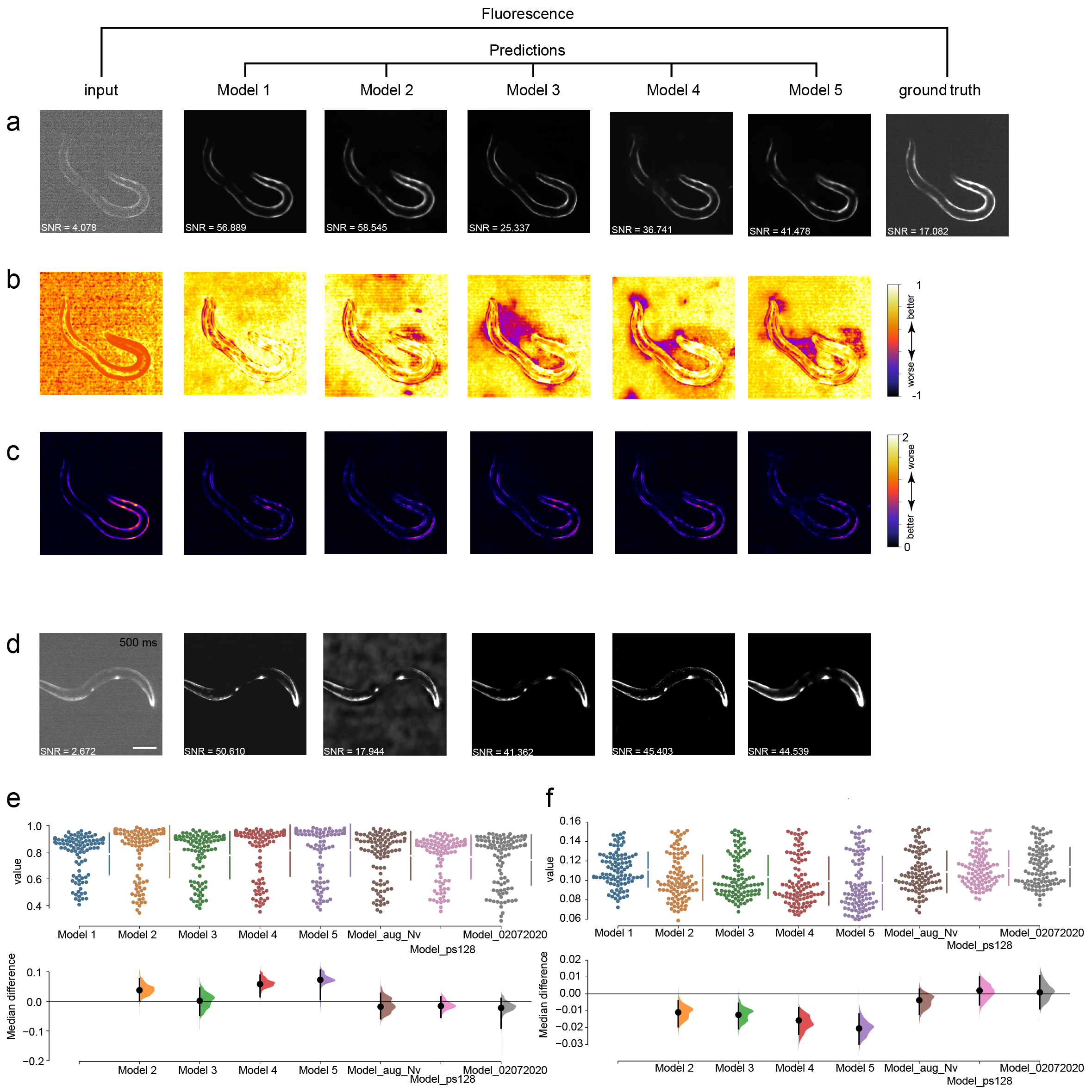

g

Model	N images	Patches per image	Augmentation	Batch size	Patch size	Percentage Validation
1	2808	10	No	64	64 x 64	10
2	2500	5	No	64	128 x 128	15
3	25000	10	Yes	64	128 x 128	10
4	2500	10	No	64	64 x 64	10
5	2500	20	No	64	64 x 64	15

Supplementary Figure 2: Benchmarking different models and their qualifiers. Caption on the next page.

Supplementary Figure 2: **continued**: **a,** CARE restorations of the input low SNR image with five best models. **b,** SSIM maps of the input image (left most image) and the different predictions compared to the ground truth, fluorescence image. **c,** NRMSE maps of the input image (left most image) and the different predictions compared to the ground truth, fluorescence image. **d,** Input bioluminescence image and the inferences of the ground truth for the different models. Same scale bar = 50 µm for all panels. **e, f,** Summary statistics of the average (e) SSIM and (f) NRMSE per image for all models, tested for N=100 different, unseen images. The median difference for 7 comparisons against the shared control Model 1 are shown in the above Cumming estimation plot. The raw data is plotted on the upper axes. On the lower axes, median differences are plotted as bootstrap sampling distributions, comparing model 1 with the rest. The dots inthe lower graph indicate the mean of the distribution of median differences. Each 95% confidence interval is indicated by the ends of the vertical error bars. For (e) The unpaired median difference between Model 1 and Model 2 is 0.0374 [95.%CI 0.0039, 0.0764] with p=0.0132; between Model 1 and Model 3 is 0.00174 [95.0%CI -0.0475, 0.0453] with p=0.95; between Model 1 and Model 4 is 0.0582 [95.0%CI 0.0155, 0.0889] with p=4e-4; between Model 1 and Model 5 is 0.0728 [95.0%CI 0.0055, 0.106] with $p<1e-12$; between Model 1 and Model_aug_Nv is -0.0182 [95.0%CI -0.0589, 0.0273] with p=0.548; between Model 1 and Model_ps128 is -0.0159 [95.0%CI -0.0542, 0.0162] with p=0.308; Model 1 and Model_02072020 is -0.0222 [95.0%CI -0.0906, 0.0103] with p=0.155. For (f) The unpaired median difference between Model 1 and Model 2 is -0.011 [95.0%CI -0.0196, -0.00407] wit a p-value 4e-4; between Model 1 and Model 3 is -0.0125 [95.0%CI -0.0208, -0.0055] with $p<1e-12$; between Model 1 and Model 4 is -0.0157 [95.0%CI -0.0241, -0.00811] with $p<1e-12$; between Model 1 and Model 5 is -0.0205 [95.0%CI -0.0299, -0.0119] with $p<1e-12$; between Model 1 and Model_aug_Nv is -0.00379 [95.0%CI -0.0119, 0.00277] with p=0.185; between Model 1 and Model_ps128 is 0.00195 [95.0%CI -0.00663, 0.0101] with p=0.8; between Model 1 and Model_02072020 is 0.000854 [95.0%CI -0.00887, 0.0107] with p=0.907. **g** Table of the hyperparameters used to train the different models.

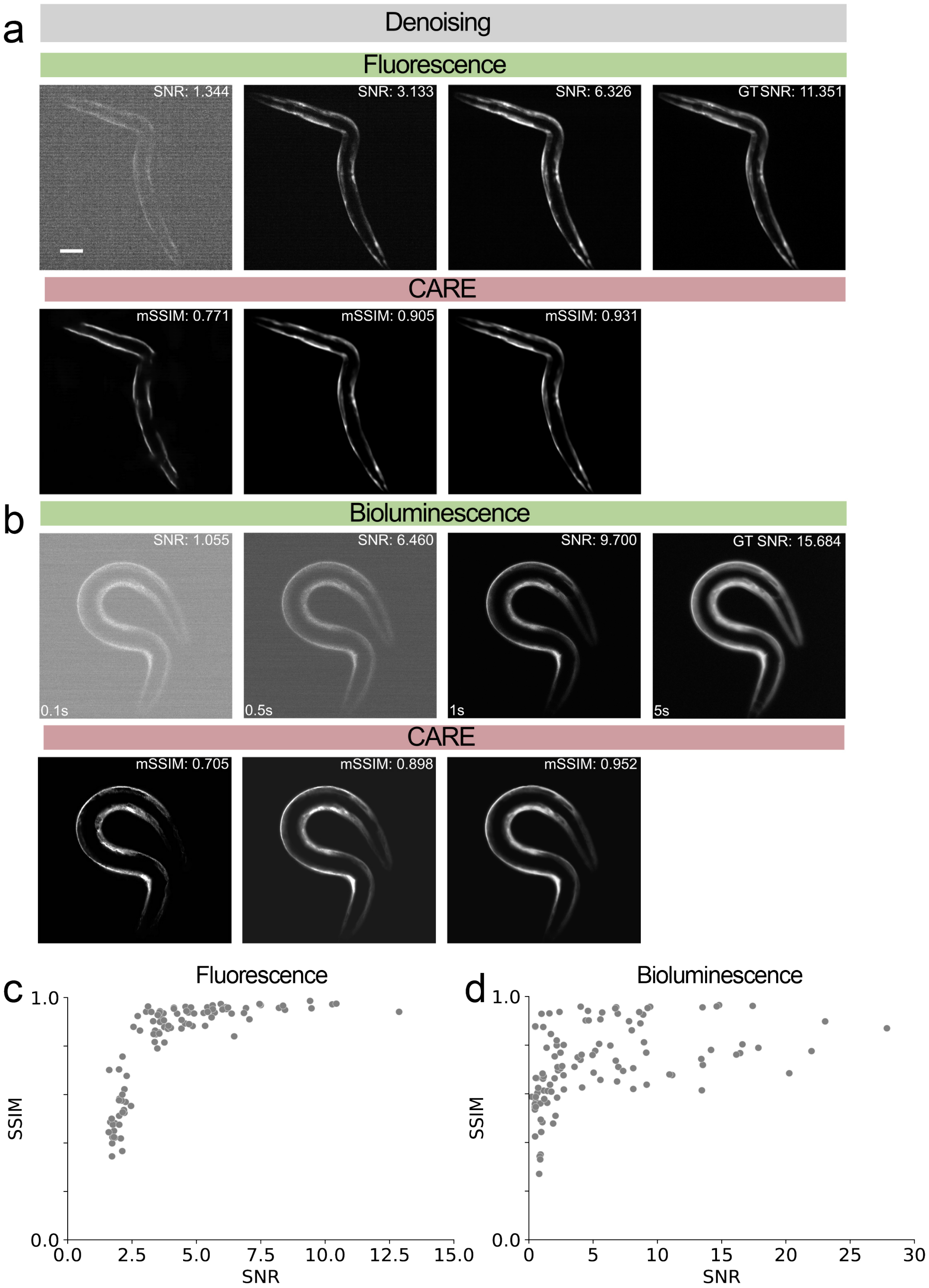

Supplementary Figure 3: Limits of restoration quality for CARE.
a,b, Example fluorescence (a) and bioluminescence (b) images with varying SNR and the corresponding CARE restoration. A larger SNR in the input image provides a better SSIM index. GT SNR = signal/noise ratio of the ground truth image.**c,d** Plot of the SSIM vs input SNR indicates a threshold for the restoration quality for (c) fluorescence and (d) bioluminescence images.

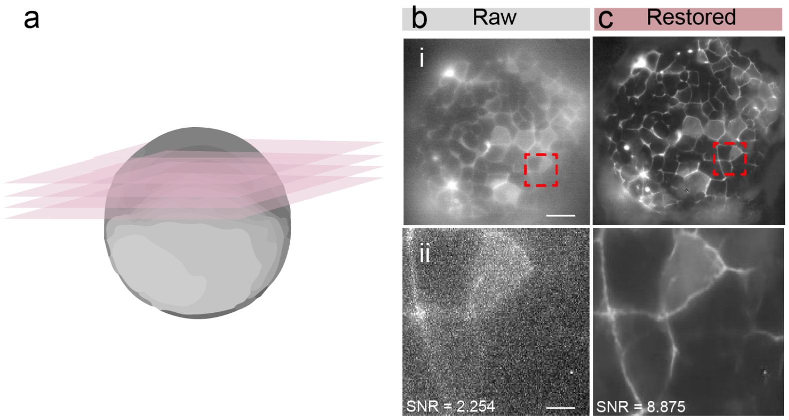

Supplementary Figure 4: CARE denoising zebrafish embryos improves edge detection
a, Schematic of the zebrafish embryo at dome stage indicating the imaging plane of the bioluminescence pictures. **b,** Raw bioluminescence image (i, Scale bar = 100µm.) and high magnification close up (ii, scale bar = 20µm). **c,** CARE restoration of the picture in b using a published model for tessellated epithelial monolayers.

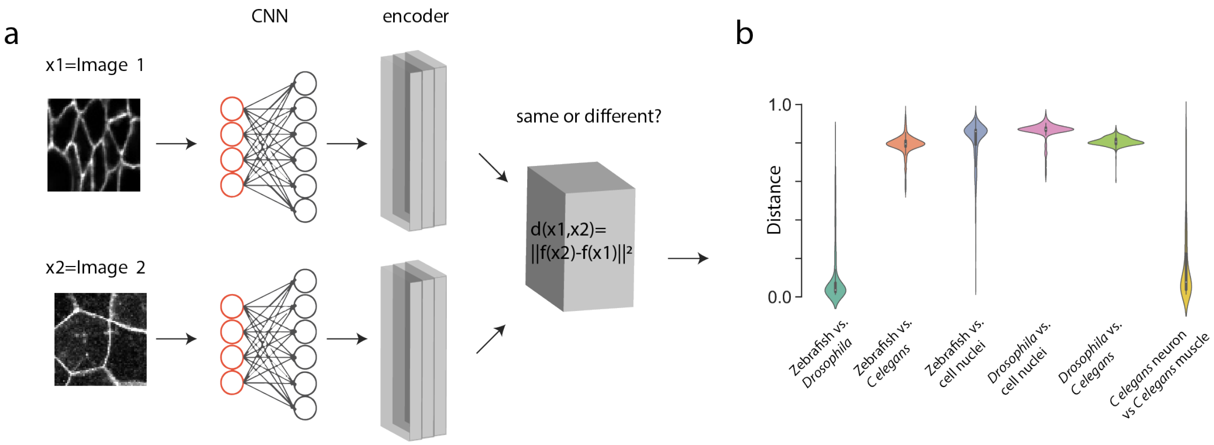

Supplementary Figure 5: Image matching with neuronal networks classification
a, Schematic of the Siamese CNN with two input images taken from a *Drosophila* and the zebrafish data set (this study). **b** Euclidean distance for different image pairs taken from various data sets used on this study, describing epithelia in *Drosophila*, zebrafish but also *C. elegans* muscles and neurons. Each violin plot was constructed from N=1000 data points (except for the last neuron vs muscle was constructed with N=563). Box indicates 50% of the data points centered around the median (whit dot). Whiskers embrace 90% of the data.

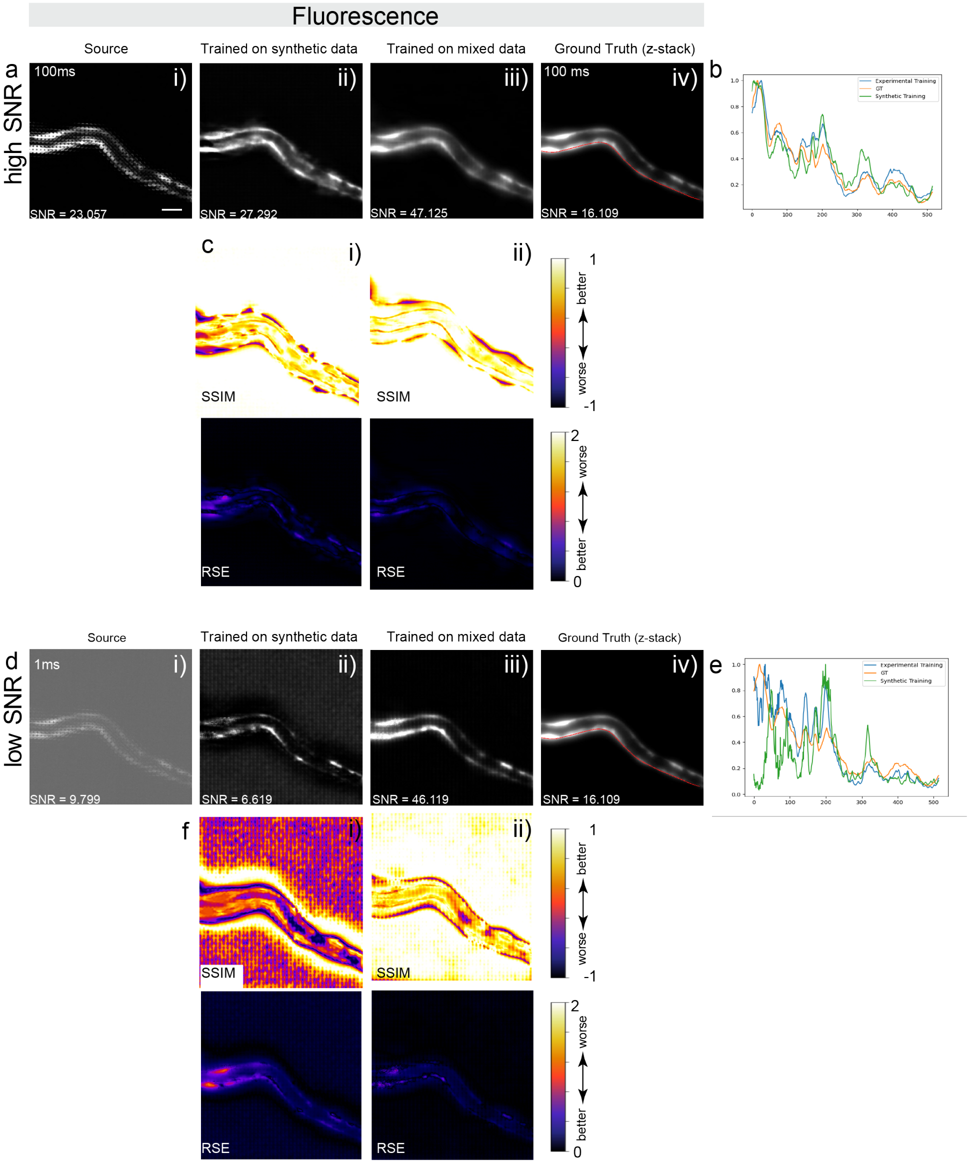

Supplementary Figure 6: Reconstruction of lightfield images preserves intensity distribution To test the quality of the training and validity of the model, whether or not the relative input intensities are preserved in the output images, reconstructions of the different models have been tested against the ground truth dataset. **a,** From left to right: i) Input fluorescence lightfield image acquired at 100 ms representing the high SNR image. Reconstruction with a model exclusively trained on ii) synthetic and iii) experimental lightfield data. iv) Maximum intensity projection of a 3D stack acquired with traditional, widefield fluorescence microscopy. Same scale bar = 50 μm for all images. **b,** Comparison of the intensity profile for the predicted LF image and the ground truth fluorescence image in the ventral muscles. **c,** SSIM and NRMSE maps of the i) synthetic and ii) mixed experimental data. **d,** From left to right: i) Input fluorescence image acquired at 1ms representing the low SNR image. Reconstruction with a model exclusively trained on ii) synthetic and iii) experimental lightfield data. iv) Maximum intensity projection of a 3D stack acquired with traditional, widefield fluorescence microscopy. Note, b) iv and d) iv is the same projection from the ground truth image stack. **e,** Comparison of the intensity profile for the predicted LF image and the ground truth fluorescence image in the ventral muscles. **f,** SSIM and NRMSE maps of the i) synthetic and ii) mixed experimental data.

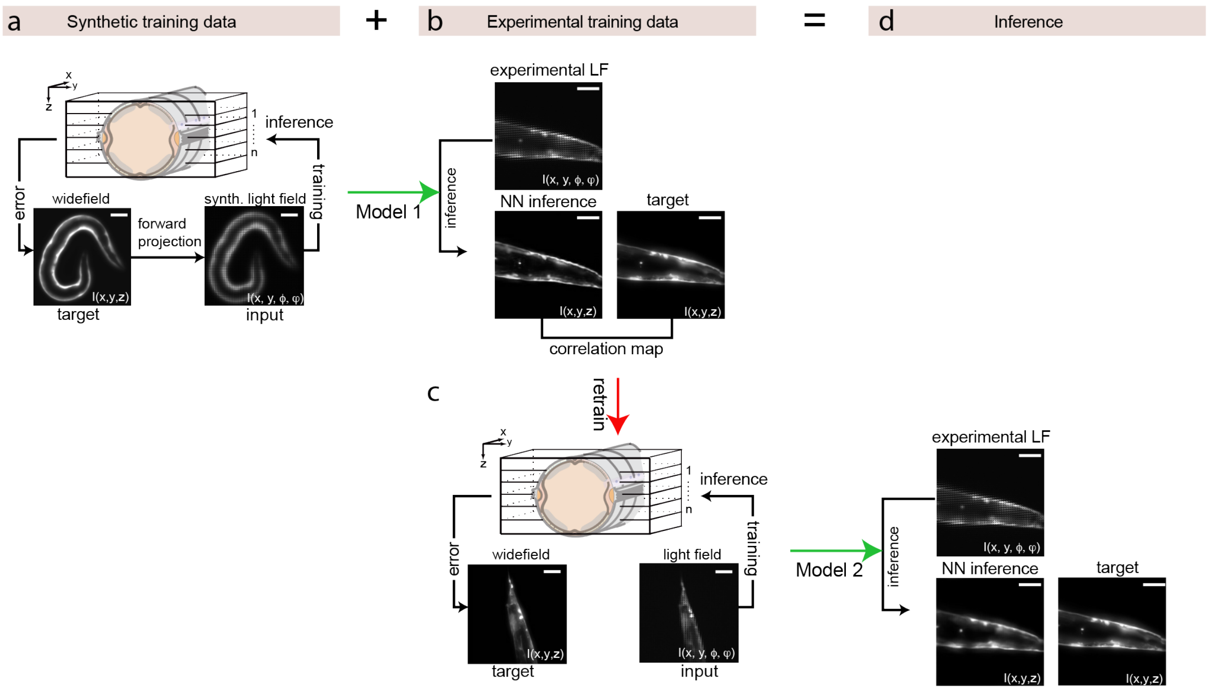

Supplementary Figure 7: Transfer learning pipeline for lightfield reconstruction
A dual training sequence employing a knowledge expansion algorithm was applied to improve the light field reconstruction quality. **a,** First, an experimental z-stack (fluorescence) was used in conjunction with synthetic light field data. The synthetic light field images were obtained from a simulated PSF of our system. Then, the common training process starts by feeding the network with the synthetic light field images and measuring the inference error against the experimental three-dimensional stacks with which we created the data. This will provide Model 1. **b** An experimental dataset, e.g. experimental three-dimensional stacks and their corresponding experimental light field images obtained from the same scene/animal and registered by finding the correlation map between the three-dimensional reconstruction and the three-dimensional experimental stack. For this, we rectify both images to ensure the matching of the pixel size and the proper alignment of the middle lenslet in the LF image. The maximum projection intensity was used to extract the structural information and find the transformation matrix to register the training pair. The registration was necessary as the two experimental data sets were acquired on two different cameras. **c,** After registration, we used the weights of Model 1 as a starting point and trained again using the aligned experimental dataset. This knowledge expansion yielded Model 2 and was applied to reconstruct experimental light field images with higher quality. **d,** Once the Model 2 is trained, predictions of unseen data achieves a higher performance than Model 1 (see also Figure 6c). Scale bar = 50μm for all images.

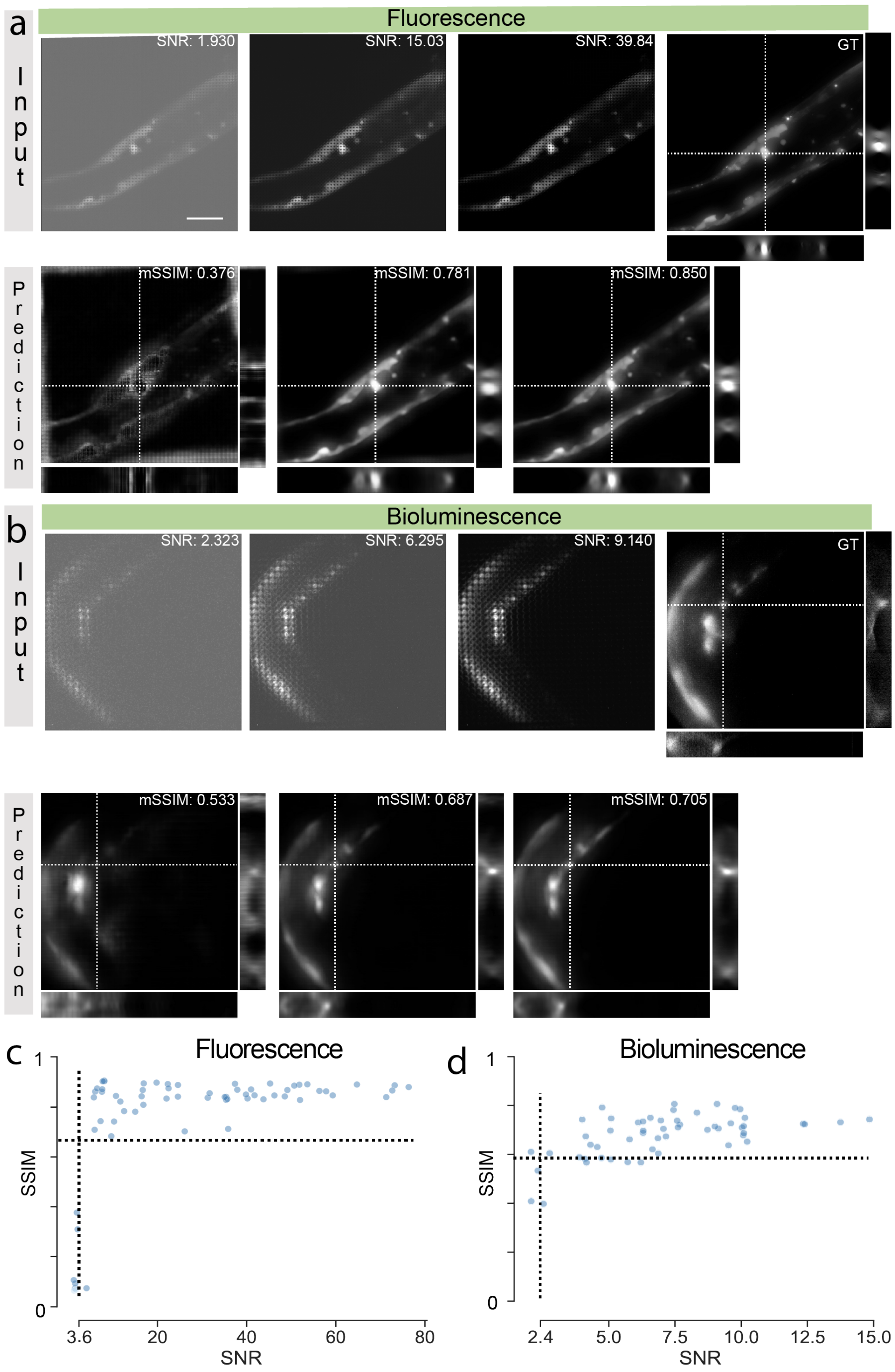

Supplementary Figure 8: Limits of restoration quality for the Lightfield reconstruction.
a,b, Example fluorescence (a) and bioluminescence (b) images with varying SNR and the corresponding light field restoration. A larger SNR in the input image provides a better SSIM index. GT=ground truth; SNR = signal/noise ratio. Scale bar = 50µm for all images. **c, d** Plot of the SSIM vs input SNR indicates a threshold for the restoration quality for (c) fluorescence and (d) bioluminescence images.

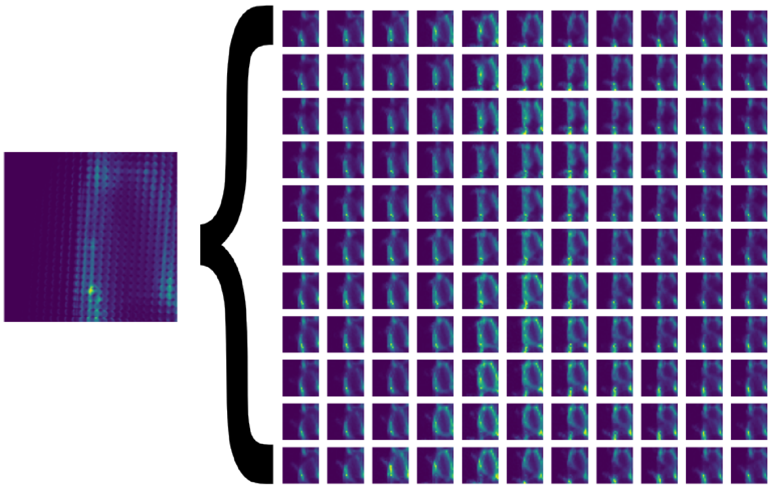

Supplementary Figure 9: Perspective extraction for lightfield reconstruction
Extracted perspective views from the bioluminescence picture derived through the microlens array.

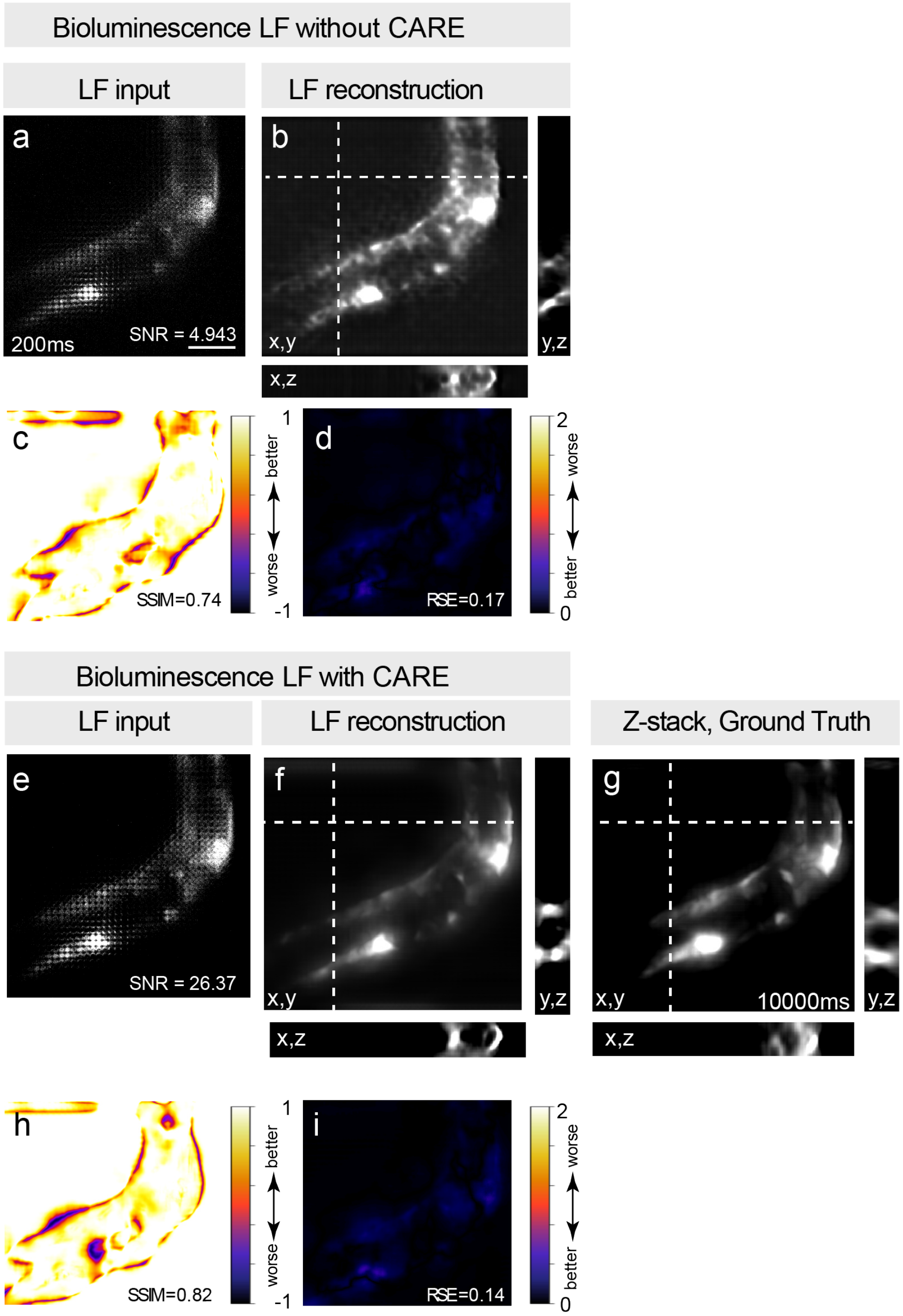

Supplementary Figure 10: CARE denoising of lightfield images improves 3D reconstructions **a,** Raw, low SNR light field image of an immobilized bioluminescent animal taken at 200ms exposure time. Same scale bar = 50 µm for all images. **b,** Reconstruction without prior denoising. **c,d** SSIM (c) and NRMSE (d) of the raw light field image tested against the 'ground truth' z-stack. **e,** Raw, high SNR light field image after CARE denoising of an immobilized bioluminescent animal taken at 200ms exposure time. **f,** Reconstruction with a priori CARE denoising. **g,** Maximum intensity projection of a 'ground truth' of z-stack of the same worm in bioluminescence contrast taken at 10s exposure time. All light field models were trained with mixed (experimental and simulated) data.**h,i** SSIM (h) and NRMSE (i) of the denoised data tested against the 'ground truth'.

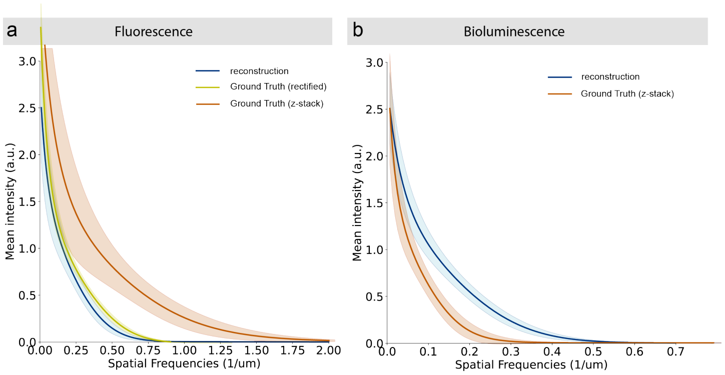

Supplementary Figure 11: Image resolution of the light field microscope
a, Fluorescence: Azimuthal average of the spatial frequencies in the FFT of the maximum intensity projection of a fluorescence image stack composed of either the ground truth before and after rectification, or the reconstructed light field image. Note, rectification includes downsampling to match the size and pixels of the light field image and thus causes a degradation of the image resolution. Importantly, the neuronal network for lightfield reconstruction was trained using the rectified ground truth, which leads to a comparable light field reconstruction. Importantly, the observed resolution of 1.5μm is 3 times better than the nominal resolution expected from the lenslet array. Mean±SD for N=9 technical replicates. **b,** Bioluminescence: Azimuthal average of the spatial frequencies in the FFT of the maximum intensity projection of a bioluminescence image stack composed of the ground truth, and the reconstructed light field image stack. Note, the 'ground truth' image stack was acquired using 10s exposure time in the bioluminescence mode, which lead to possible image artefacts (movement, degraded SNR), leading to a unwanted degradation of the spatial resolution. Mean±SD for N=10 technical replicates.

Supplementary Movies

Supplementary Movie 1 Dynamics of the DAF-16 transcription factor exclusively in muscles and neurons in response to external heat. For display purposes, the video was denoised using the deep learning pipelines developed in this manuscript. Scale bar = 50 µm.

Supplementary Movie 2 Dynamics of mouse embryonic stem cells within a spheroid. For display purposes, the video was denoised using the deep learning pipelines developed in this manuscript. During imaging, spheroids were continuously perfused with fresh cofactor. Subtle variations in cofactor bioavailability and tissue distribution lead to transient intensity fluctuations. Scale bar = 100 µm.

Supplementary Movie 3 Three dimensional calcium dynamics of a freely moving animal. The video was denoised and reconstructed from a 2D lightfield image using the deep learning pipelines developed in this manuscript. Scale bar = 100 µm.
