# Supplementary material for: Volumetric imaging of fast cellular dynamics with deep learning enhanced bioluminescence microscopy
Source: Commun Biol. 2022 Dec 3;5:1330. doi: 10.1038/s42003-022-04292-x (PMC9719505; doi:10.1038/s42003-022-04292-x)
Supplement: Supplementary file 3 — Description of Additional Supplementary Files [file 42003_2022_4292_MOESM3_ESM.pdf]

Description of Additional Supplementary Files

File name: Supplementary Movie 1
Description: Dynamics of the DAF-16 transcription factor exclusively in muscles and neurons in response to external heat. For display purposes, the video was denoised using the deep learning pipelines developed in this manuscript. Scale bar = 50 µm.

File name: Supplementary Movie 2
Description: Dynamics of mouse embryonic stem cells within a spheroid. For display purposes, the video was denoised using the deep learning pipelines developed in this manuscript. During imaging, spheroids were continuously perfused with fresh cofactor. Subtle variations in cofactor bioavailability and tissue distribution lead to transient intensity fluctuations. Scale bar = 100 µm.

File name: Supplementary Movie 3
Description: Three dimensional calcium dynamics of a freely moving animal. The video was denoised and reconstructed from a 2D lightfield image using the deep learning pipelines developed in this manuscript. Scale bar = 100 µm.

File name: Supplementary Data 1
Description: Source Data of all the graphs in the main and supplementary figures.
