# Supplementary material for: Volumetric imaging of fast cellular dynamics with deep learning enhanced bioluminescence microscopy
Source: Commun Biol. 2022 Dec 3;5:1330. doi: 10.1038/s42003-022-04292-x (PMC9719505; doi:10.1038/s42003-022-04292-x)
Supplement: Supplementary file 8 — Reporting Summary [file 42003_2022_4292_MOESM8_ESM.pdf]

nature portfolio

Corresponding author(s): Michael Krieg

Last updated by author(s): Nov 12, 2022

Reporting Summary

Nature Portfolio wishes to improve the reproducibility of the work that we publish. This form provides structure for consistency and transparency in reporting. For further information on Nature Portfolio policies, see our Editorial Policies and the Editorial Policy Checklist.

Statistics

For all statistical analyses, confirm that the following items are present in the figure legend, table legend, main text, or Methods section.

n/a	Confirmed	
☐	☒	The exact sample size (*n*) for each experimental group/condition, given as a discrete number and unit of measurement
☐	☒	A statement on whether measurements were taken from distinct samples or whether the same sample was measured repeatedly
☒	☐	The statistical test(s) used AND whether they are one- or two-sided<br>*Only common tests should be described solely by name; describe more complex techniques in the Methods section.*
☒	☐	A description of all covariates tested
☒	☐	A description of any assumptions or corrections, such as tests of normality and adjustment for multiple comparisons
☒	☐	A full description of the statistical parameters including central tendency (e.g. means) or other basic estimates (e.g. regression coefficient) AND variation (e.g. standard deviation) or associated estimates of uncertainty (e.g. confidence intervals)
☒	☐	For null hypothesis testing, the test statistic (e.g. *F*, *t*, *r*) with confidence intervals, effect sizes, degrees of freedom and *P* value noted<br>*Give P values as exact values whenever suitable.*
☒	☐	For Bayesian analysis, information on the choice of priors and Markov chain Monte Carlo settings
☐	☒	For hierarchical and complex designs, identification of the appropriate level for tests and full reporting of outcomes
☒	☐	Estimates of effect sizes (e.g. Cohen's *d*, Pearson's *r*), indicating how they were calculated

Our web collection on statistics for biologists contains articles on many of the points above.

Software and code

Policy information about availability of computer code

Data collection	Python 3.7.0, Micromanager 2.0 gamma, Pycromanager 0.17.10
Data analysis	ImageJ 2.3.0, CSBDeep-CARE 0.5.2, Tensorflow 1.15.0, VCD-Net 10.22.2020, Python 3.7.0, Matlab R2021a, keras 2.0.0

For manuscripts utilizing custom algorithms or software that are central to the research but not yet described in published literature, software must be made available to editors and reviewers. We strongly encourage code deposition in a community repository (e.g. GitHub). See the Nature Portfolio guidelines for submitting code & software for further information.

Data

Policy information about availability of data

All manuscripts must include a data availability statement. This statement should provide the following information, where applicable:

- Accession codes, unique identifiers, or web links for publicly available datasets
- A description of any restrictions on data availability
- For clinical datasets or third party data, please ensure that the statement adheres to our policy

The code of the training and inference pipelines and instructions to run it will be freely available under this link: https://gitlab.icfo.net/NMSB/efmicro
Upon publication we will deposit all training data to zenodo.org

Human research participants

Policy information about studies involving human research participants and Sex and Gender in Research.

Reporting on sex and gender	N/A
Population characteristics	N/A
Recruitment	N/A
Ethics oversight	N/A

Note that full information on the approval of the study protocol must also be provided in the manuscript.

Field-specific reporting

Please select the one below that is the best fit for your research. If you are not sure, read the appropriate sections before making your selection.

☒ Life sciences ☐ Behavioural & social sciences ☐ Ecological, evolutionary & environmental sciences

For a reference copy of the document with all sections, see nature.com/documents/nr-reporting-summary-flat.pdf

Life sciences study design

All studies must disclose on these points even when the disclosure is negative.

Sample size	We choose the training, validation and testing datasets size to be representative of the variability seen across the time-points.
Data exclusions	No data was excluded from the manuscript.
Replication	For all experiments we provide the code and training data to retrain all used models and reproduce the findings.
Randomization	For each experiment, every sample was randomly selected from the pre-shuffled dataset.
Blinding	The models were optimized by based on a random validation set taken from the main dataset that had no overlap neither with the batch being processed for training nor the batch for testing.

Reporting for specific materials, systems and methods

We require information from authors about some types of materials, experimental systems and methods used in many studies. Here, indicate whether each material, system or method listed is relevant to your study. If you are not sure if a list item applies to your research, read the appropriate section before selecting a response.

Materials & experimental systems

n/a	Involved in the study	
☒	☐	Antibodies
☐	☒	Eukaryotic cell lines
☒	☐	Palaeontology and archaeology
☐	☒	Animals and other organisms
☒	☐	Clinical data
☒	☐	Dual use research of concern

Methods

n/a	Involved in the study	
☒	☐	ChIP-seq
☒	☐	Flow cytometry
☒	☐	MRI-based neuroimaging

Eukaryotic cell lines

Policy information about cell lines and Sex and Gender in Research

Cell line source(s)	Mouse Embryonic Stem Cell (mESC) line was generated by crossing a female 129 and male C57BL/6 mice. mESC G4, F1 hybrid was chosen due to superior developmental potential according to George et al. (2006) PNAS.
Authentication	No authentication was used.

Mycoplasma contamination	Cells were monthly checked for mycoplasma contamination using PCR amplification.
Commonly misidentified lines (See ICLAC register)	None.

Animals and other research organisms

Policy information about studies involving animals; ARRIVE guidelines recommended for reporting animal research, and Sex and Gender in Research

Laboratory animals	C. elegans, Zebrafish
Wild animals	N/A
Reporting on sex	Hemaphrodites
Field-collected samples	Zebrafish embryos were kept in E3 medium at between 25° and 31°C before experiments and staged according to morphological criteria and hours post fertilization (hpf)
Ethics oversight	All protocols used have been approved by the Institutional Animal Care and Use Ethic Committee (PRBB–IACUEC) and implemented according to national and European regulations.

Note that full information on the approval of the study protocol must also be provided in the manuscript.
